# A Non-invasive Imaging Approach to Evaluating the Immediate Mattifying Effects of Topical Skin Care Products: Method Standardization and Validation

**DOI:** 10.7759/cureus.91752

**Published:** 2025-09-06

**Authors:** Maheshvari N Patel, Nayan Patel, Apeksha Merja

**Affiliations:** 1 Clinical Research, NovoBliss Research Private Limited, Ahmedabad, IND; 2 Pharmacology, Swaminarayan University, Ahmedabad, IND; 3 Clinical Research Operations, NovoBliss Research Private Limited, Ahmedabad, IND

**Keywords:** cosmetics, image analysis, mattifying effect, skin pores, skin roughness, skin smoothness

## Abstract

Immediate mattifying effects and pore-minimizing claims are common in facial cosmetics; however, objective and standardized assessment methods remain underreported. This in-house exploratory evaluation aimed to standardize methodology for assessing the reduction in facial pores by using instrumental assessment and image analysis. A split-face design was conducted on 20 healthy female volunteers. A cosmetic leave-on face cream was applied to the right side (treated), while the left side served as an untreated control. Skin roughness, smoothness, and pore size were assessed using the VISIOSCAN VC 20 Plus (Courage+Khazaka electronic GmbH, Cologne, Germany). Pore size and pore area were further evaluated using Image-Pro® 10.0 (Media Cybernetics, Inc., Rockville, Maryland, United States), at baseline and 15 minutes post-application. Application of the test product at the treated site resulted in a measurable reduction in both skin pore size and skin pore area, accompanied by significant improvements in skin surface roughness and smoothness within 15 minutes post application. In contrast, the control site (water-rinse) did not exhibit any notable changes across these parameters. These differences highlight the sensitivity of the analytical approach in detecting early, product-induced skin surface improvements. This in-house exploratory evaluation primarily served to assess the feasibility and robustness of a standardized methodology for evaluating the immediate effects of cosmetic leave-on formulations. By combining high-resolution photographic image analysis with VISIOSCAN VC 20 Plus instrumentation, the study established a reproducible, quantifiable approach for assessing short-term changes in skin surface attributes such as pore size, roughness, and smoothness.

## Introduction

Skin pores are defined as the visible superficial openings of pilosebaceous units, predominantly located on the face, which play a crucial role in thermoregulation and sebum secretion. Their size and appearance can be influenced by multiple intrinsic and extrinsic factors, including chronological aging, genetic predisposition, sex-related hormonal variations, ethnicity, photodamage from chronic ultraviolet (UV) exposure, and sebaceous gland activity. [1] Several studies have reported that, with advancing age, there is a notable increase in the number and total surface area of facial pores, particularly in regions such as the nasal area and the medial aspects of the cheeks. This phenomenon is believed to be associated with age-induced changes in dermal elasticity, collagen degradation, and altered sebum dynamics, contributing to more prominent pore visibility and textural irregularities of the skin surface [2,3].

Visible facial pores are a prevalent cosmetic concern, particularly among individuals with oily or combination skin types. These features are often associated with increased sebum production and reduced skin elasticity, contributing to an uneven skin texture and enlarged pore appearance [4]. The application of mattifying face creams aims to address these concerns by reducing skin shine and minimizing the appearance of pores. Evaluating the immediate efficacy of such products requires objective, non-invasive, and reproducible assessment methods.

Advancements in imaging technologies, including two-dimensional (2D) and three-dimensional (3D) image analysis systems, have facilitated the quantitative evaluation of skin surface characteristics, enabling precise measurement of parameters like pore size, density, and skin roughness [5]. To assess such effects, non-invasive imaging tools like the VISIOSCAN VC 20 Plus (Courage+Khazaka electronic GmbH, Cologne, Germany) have been widely used to evaluate skin surface texture characteristics, including smoothness, roughness, under controlled lighting conditions [6]. Similarly, image analysis using software such as Image-Pro® (Media Cybernetics, Inc., Rockville, Maryland, United States) allows for the objective quantification of parameters like pore size and distribution.

This technical evaluation was conducted for the preliminary assessment of the immediate mattifying potential of a cosmetic face cream, specifically its effects on pore visibility and skin surface texture. The primary objective was to evaluate changes in pore size using image analysis software and to examine alterations in skin texture and shine through non-invasive imaging techniques following a single-time application.

## Technical report

Materials and methods

Design and Method Standardization

This was a double-blind, randomized study with a split-face design, where the right cheek was designated as the treated site (application of the test product), and the left cheek served as the untreated control (subjected only to water rinse). The evaluation was performed on 20 healthy female volunteers, aged 25-45 years, with no known dermatological conditions.

This exploratory evaluation was conducted on April 16, 2025 (at 24.8°C and 45% humidity), at NovoBliss Research Private Limited (CRO), Ahmedabad, India, with the objective of standardizing a methodology for assessing the immediate mattifying effect of a topical face cream formulation. This split-face assessment was designed to refine image analysis techniques and instrument performance under controlled ambient conditions, with findings intended for internal discussion and future study planning.

Ethical Consideration

This evaluation was conducted to assess the technical feasibility of imaging procedures and to generate preliminary clinical observations related to the effects of a topical face cream. The study was performed on a small group of participants to evaluate changes in skin texture and pore visibility using standardized, non-invasive assessment tools. All participants were briefed on the study procedures, and written informed consent was obtained prior to enrollment, ensuring ethical compliance and voluntary participation. 

Inclusion and Exclusion Criteria

Participants were selected from in-house study staff and were required to meet specific inclusion criteria to ensure consistency in skin condition and test outcomes. Eligible individuals were female adults aged 25-45 years, in generally good health, and free from any active dermatological conditions on the face. Participants were required to have no recent history of cosmetic procedures or the use of topical treatments (e.g., retinoids, corticosteroids, or exfoliating agents) within the previous two weeks. All participants provided informed consent and agreed to follow the testing protocols.

Exclusion criteria included individuals with known hypersensitivities or allergies to cosmetic products or ingredients, as well as those with current skin infections, inflammation, or open wounds in the facial area. Participants who were pregnant, breastfeeding, or undergoing any systemic medication that could influence skin condition were also excluded. To minimize variability, individuals with facial tattoos, scars, or excessive facial hair that could interfere with image analysis were not enrolled. Additionally, participants who had undergone aesthetic treatments such as chemical peels, microdermabrasion, or laser resurfacing within the past month were excluded from the study.

Test Product

The test product used in this method standardization was a topical face cream. Prior to product application, all participating study staff were instructed to rinse their face thoroughly with water and gently pat dry using a clean muslin cloth to ensure a consistent skin surface condition. A pea-sized quantity of the test cream was applied evenly on the right side of the face (treated site) using the fingertips, following a standardized method to ensure uniform coverage. The left side of the face served as the untreated control and was rinsed only with water, as per the split-face study design.

Study Procedure and Assessment

Volunteers abstained from applying any cosmetic or skincare products on the day of assessment to minimize variability. All assessments were conducted under standardized environmental conditions. Light intensity was measured using a Lux meter at five fixed points within the designated digital photography area (four corners and the center of the room), both prior to initiation and following completion of each photography session. Readings were taken 10 minutes after switching on the lights to allow stabilization, and the acceptable range was maintained at 280-330 Lux, with a permissible variation of ±50 Lux from the daily reference value. Imaging was performed using a Nikon D3300 digital camera (Nikon Corporation, Shinagawa, Tokyo, Japan) with a 25 mm lens, positioned one foot away from the subject and aligned at a 90° angle to the site. These controls ensured consistency in ambient lighting and photographic capture conditions across all participants.

For pore analysis, pore count and area were quantified from the images using Image-Pro Software (version 10), which employed a 2D filtering algorithm to enhance contrast for accurate pore detection. Simultaneously, non-invasive skin surface imaging was carried out using the VISIOSCAN VC 20 Plus, which utilizes ultraviolet A light to evaluate surface parameters including skin roughness, smoothness, and pore visibility.

All assessments were performed under controlled ambient temperature (at 24.8°C and 45% humidity) to ensure consistency. This dual-instrument approach allowed for a comparative assessment of skin textural changes and pore characteristics, supporting internal method standardization for future controlled evaluations.

**Figure 1:**
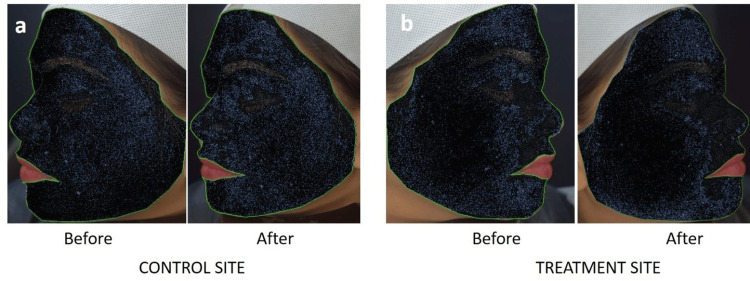
Assessment of pore size and pore count for (a) control site (left side of face) (b) treatment site (right side of face) with Image-Pro® 10.0* *Media Cybernetics, Inc., Rockville, Maryland, United States

Image-Pro assessment methods for pore size reduction: High-resolution facial images were captured using a Nikon D3300 digital camera with a 25 mm lens under standardized ambient lighting and fixed positioning to maintain consistency across all participants. Images from both the treated (right) and control (left) sides of the face were taken at two time points: baseline and 15 minutes post application of the face cream. The photographs were analyzed using Image-Pro 10.0. A consistent region of interest (ROI) on the cheek area was selected for each subject. The analysis employed a 2D filter to enhance the contrast of pore structures, allowing for clearer segmentation and identification. The software automatically computed pore count and mean pore area (pixel²) within the region of interest. These parameters were used to evaluate the immediate effect of the leave-on cosmetic product on pore visibility and skin surface uniformity (Figure 1).

Skin texture assessment via VISIOSCAN VC 20 Plus: Prior to the assessment, study participants were acclimatized under controlled ambient conditions for 15 minutes to stabilize skin surface characteristics. Skin topography, including surface roughness, smoothness, and pore characteristics, was evaluated using the VISIOSCAN VC 20 Plus, a non-invasive optical device that utilizes UVA light to quantitatively analyze the skin surface, enabling objective assessment of short-term skin surface changes attributable to the test formulation.

The VisioScan VC 20 Plus instrument used in this study has specific measurement principles where the interpretation of values is inverse to the clinical perception. For skin roughness, an increase in the numeric value measured by VisioScan VC 20 Plus corresponds to a reduction in clinically perceived roughness. Thus, higher values indicate smoother skin surfaces. For skin smoothness, a reduction in the numeric value corresponds to an increase in clinical smoothness. In other words, lower numeric values reflect smoother and more refined skin texture.

Results 

Change in Facial Pores Count: Image-Pro Software

A quantitative analysis of skin pore characteristics was performed using Image-Pro image analysis software. At the control site (water rinse), the baseline mean pore count was 27783.50 ± 9015.44, which increased to 27872.15 ± 9519.76 after 15 minutes of cleansing, representing a 0.12% increase from baseline. This change was statistically non-significant (p > 0.05). In contrast, application of the test product resulted in a baseline mean pore count of 27384.60 ± 9077.51, which decreased to 24751.05 ± 8347.67 after 15 minutes post-application. This reflects a -9.89% reduction in pore count from baseline, with a highly significant difference (p < 0.0001). These results indicate that while water rinse slightly increased the visible pore count, the test formulation produced a statistically significant reduction. This suggests a potential pore-minimizing effect of the test product, supporting its efficacy in improving skin surface appearance by reducing visible pores.

Change in Pore Area: Image-Pro Software

Quantitative analysis of mean facial pore area was conducted using Image-Pro image analysis software. At the treatment site (application of the test product), the baseline mean pore area was 12.04 ± 3.44 arbitrary units, which decreased to 11.46 ± 3.81 after 15 minutes. This corresponds to a change from baseline (CFB) of -8.00%, with a highly statistically significant p-value (p < 0.0001). In contrast, at the control site (water application only), the baseline mean pore area was 12.41 ± 3.94, which increased to 12.87 ± 3.70 after 15 minutes, resulting in a CFB of +7.58%. These results indicate that short-term application of the test formulation led to a statistically significant reduction in visible pore area, whereas water rinse alone was associated with an increase. The observed decrease in pore size supports the potential pore-refining and skin-smoothing properties of the test product.

**Figure 2:**
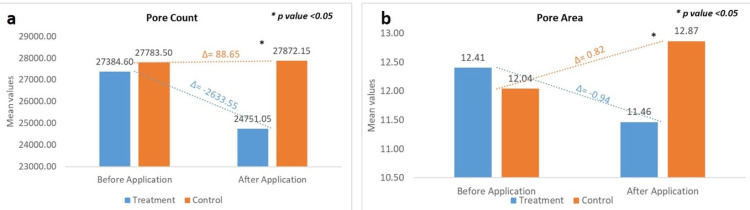
Change in (a) pore count and (b) pore area, assessed by Image-Pro® 10.0* *Media Cybernetics, Inc., Rockville, Maryland, United States

Figure 2 shows the changes in pore count and area as analysed by Image Pro.

Change in Skin Roughness and Pore Size: VISIOSCAN VC 20 Plus

Quantitative assessment of skin surface roughness was performed at the treatment site (test product application). The baseline mean roughness value was 2.14 ± 1.27, which significantly increased to 8.54 ± 3.08 after 15 minutes. This corresponds to a CFB of +422.76%, with a highly statistically significant p-value of 0.0001. At the control site (water application), the baseline mean roughness was 1.79 ± 1.20, which slightly increased to 1.89 ± 1.23 after 15 minutes, yielding a CFB of +13.85%, which was not statistically significant (p > 0.05). These findings demonstrate that, based on the measurement principles of VisioScan VC 20 Plus, the observed increase in roughness values (indicating clinically smoother skin) confirms the textural refinement effects of the test product compared to water rinse. The significant increase in surface texture parameters suggests a potential mattifying effect of the test formulation. 

Pore size was also analysed. At the treatment site (test product application), the baseline mean pore size value was 0.03 ± 0.01, which reduced after application, while at the control site, the baseline mean pore size value was 0.03 ± 0.01, which remained the same after the water rinse.

**Figure 3:**
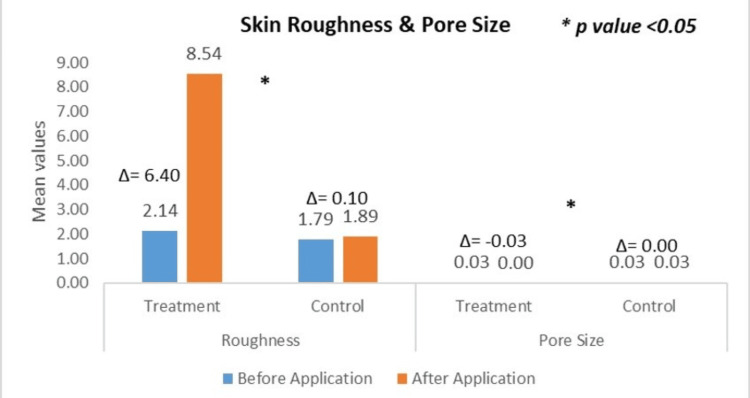
Change in (a) skin roughness and (b) pore size, assessed by VISIOSCAN VC 20 Plus * *Courage+Khazaka electronic GmbH, Cologne, Germany

Figure 3 shows the changes in roughness and pore size, as analysed with VISIOSCAN VC 20 Plus.

*Change in Skin Smoothness: VISIOSCAN VC 20 Plus* 

**Figure 4:**
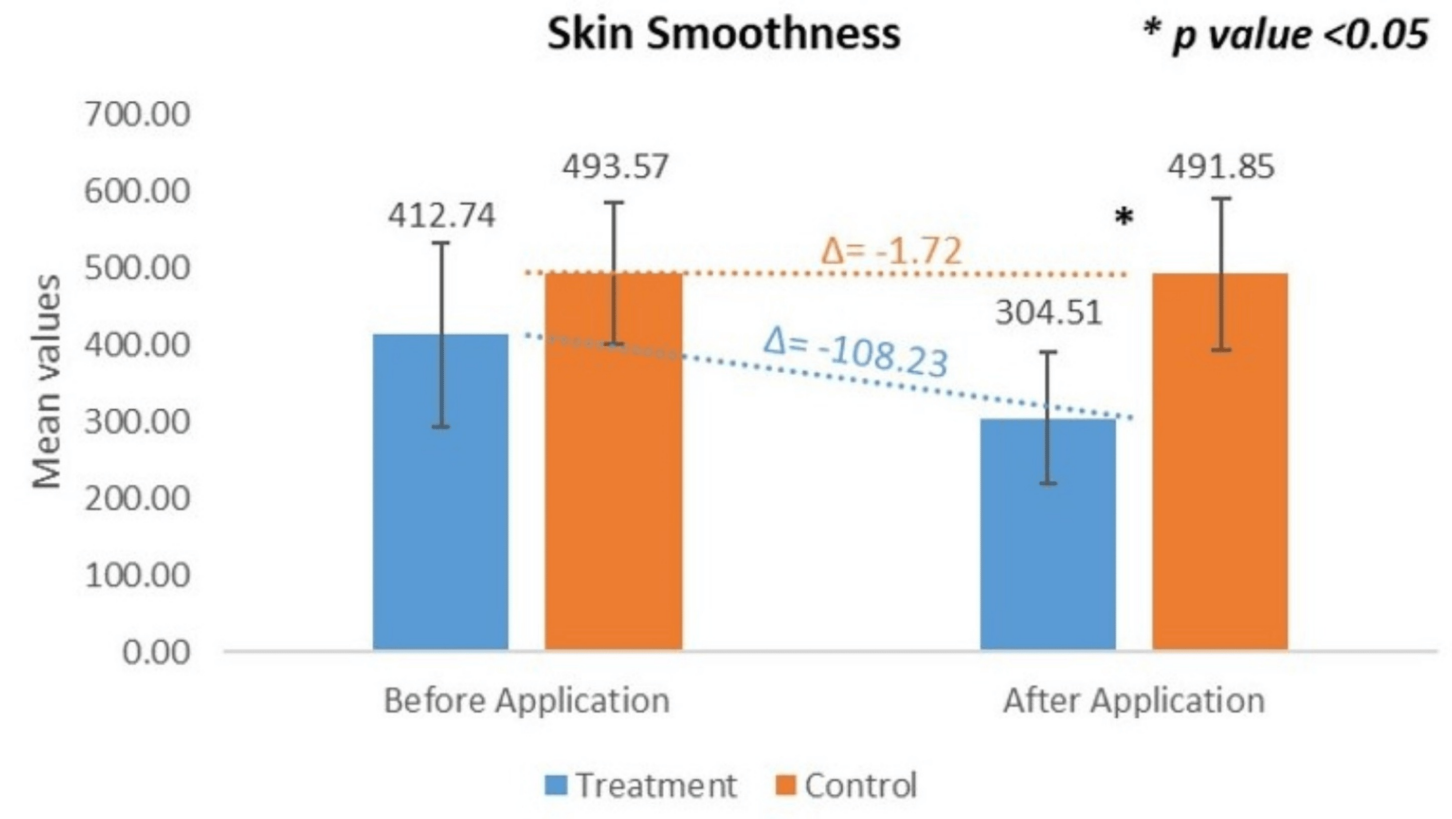
Change in skin smoothness assessed by VISIOSCAN VC 20 Plus* *Courage+Khazaka electronic GmbH, Cologne, Germany

Quantitative evaluation of skin surface smoothness was conducted at the treatment site (test product application). The baseline means smoothness value was 412.74 ± 119.66, which significantly decreased to 304.51 ± 85.16 after 15 minutes. This corresponds to a CFB of -23.61%, with a highly statistically significant p-value of > 0.001). At the control site (water rinse), the baseline mean smoothness was 493.57 ± 92.19, which slightly decreased to 491.85 ± 97.78 after 15 minutes, resulting in a CFB of -0.14%, which was not statistically significant, p > 0.05. These findings demonstrate that, based on the measurement principles of VisioScan VC 20 Plus, the observed reduction in smoothness values (indicating improved smoothness) confirms the skin-smoothing effects of the test product compared to water rinse (Figure 4)*.*

Change in Skin Wrinkles: VISIOSCAN VC 20 Plus 

**Figure 5:**
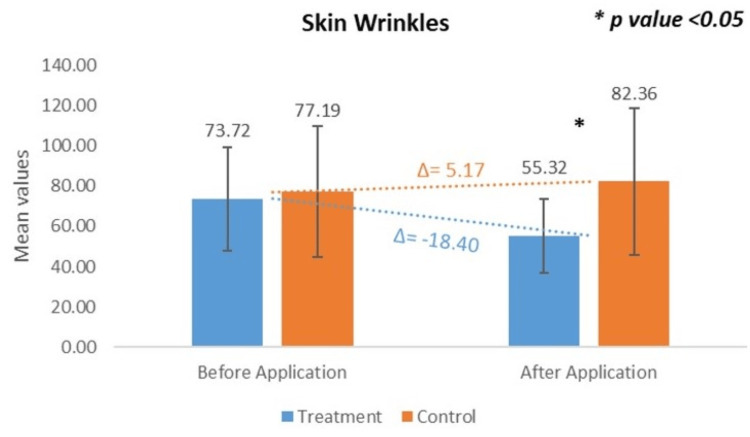
Change in skin wrinkles assessed by VISIOSCAN VC 20 Plus* *Courage+Khazaka electronic GmbH, Cologne, Germany

Quantitative evaluation of skin wrinkles conducted at the control site showed the baseline mean wrinkle value was 77.19 ± 32.56, which increased to 82.36 ± 36.38 after 15 minutes. This corresponds to a CFB of +5.70%, which was statistically significant (p < 0.05). In contrast, at the treatment site (test product application), the baseline mean wrinkle value was 73.72 ± 25.76, which decreased to 55.32 ± 18.31 after 15 minutes. This represents a CFB of -23.59%, also statistically significant (p < 0.0001). These findings indicate divergent responses at the treated and control sites. The slight increase in wrinkle values at the treated site may reflect temporary textural changes or product deposition on the skin surface, while the reduction observed at the control site could be attributed to hydration from water rinse alone. Further investigation is warranted to determine the underlying cause of these short-term effects and to evaluate their relevance over longer durations (Figure 5)*. *

## Discussion

This internal exploratory evaluation was designed to standardize assessment methodologies for evaluating the immediate mattifying effect of a cosmetic leave-on product using both photographic image analysis and non-invasive skin imaging techniques. The split-face design enabled comparative observation within the same subject, minimizing inter-individual variability.

Image-Pro-based photographic analysis demonstrated a noticeable reduction in visible pore area on the treated side 15 minutes post-application, suggesting a rapid pore-minimizing and mattifying effect potentially attributable to the occlusive or light-diffusing components of the formulation. In contrast, no change in pore size was observed on the untreated control side, indicating that the effect was specific to the test product. Such findings align with previous studies where topical formulations containing absorbents, silicones, or polymer matrices were demonstrated to deliver immediate improvements in skin texture and visual pore refinement through oil absorption and surface light scattering [7].

Complementary evaluation using the VISIOSCAN VC 20 Plus further supported the observed instrumental changes. Post-application measurements from the treated site showed improved skin smoothness and surface roughness values, likely attributable to the occlusive and film-forming properties of the formulation. In contrast, the control side did not exhibit a comparable trend in roughness and smoothness parameters, suggesting the leave-on product's potential to enhance optical skin uniformity. Additionally, a reduction in VISIOSCAN-assessed pore size on the treated side was observed, further indicating the formulation's short-term pore-minimizing effect under the conditions of this preliminary methodological assessment.

Improved skin smoothness was positively correlated with the mattifying effect observed in the study. As surface irregularities decreased, the skin reflected light more diffusely, resulting in reduced visible shine and a more uniform, matte appearance. This supports the product’s ability to enhance skin texture while delivering a mattifying benefit.

Previous studies have validated the use of the VISIOSCAN for quantifying cosmetic efficacy on skin topography, noting its ability to detect subtle changes in micro-relief patterns and pore morphology [8,9]. These preliminary findings suggest that VISIOSCAN is a suitable tool for rapid, real-time evaluation of short-term cosmetic benefits and can be integrated into early-stage product assessment.

While the current exploratory evaluation provides valuable preliminary insights into the short-term effects of the test formulation, several limitations should be acknowledged. The study was conducted on a small sample size, which limits the ability to generalize the findings. Additionally, participants were not selected based on skin type diversity, which may influence the skin’s response to treatment. The inclusion of a broader demographic range, including varied skin types and both male and female participants, would strengthen future analyses. Despite these limitations, the standardized, image-based methodology offers a promising framework for objective skin assessment. Future studies involving larger, more diverse populations and extended observation periods are recommended to validate and expand upon these findings.

## Conclusions

This in-house exploratory evaluation demonstrates the practical utility of integrating photographic image analysis with VISIOSCAN VC 20 Plus instrumentation to assess the immediate effects of a cosmetic leave-on formulation. The use of a split-face design allowed for controlled, intra-individual comparison, effectively minimizing variability and enhancing the reliability of observed differences.

Short-term application of the test product resulted in measurable improvements in skin surface parameters, including increased smoothness, reduced roughness, and a decrease in visible pore area, relative to the water rinse control site. Although these findings are not intended to establish clinical efficacy, they provide valuable methodological insights for internal benchmarking and future study design. 
